# The Intensive Diet and Exercise for Arthritis (IDEA) trial: design and rationale

**DOI:** 10.1186/1471-2474-10-93

**Published:** 2009-07-28

**Authors:** Stephen P Messier, Claudine Legault, Shannon Mihalko, Gary D Miller, Richard F Loeser, Paul DeVita, Mary Lyles, Felix Eckstein, David J Hunter, Jeff D Williamson, Barbara J Nicklas

**Affiliations:** 1 Department of Health and Exercise Science, Wake Forest University, Winston-Salem, NC, USA; 2 Department of Public Health Sciences, Wake Forest University School of Medicine, Winston-Salem, NC, USA; 3 Department of Internal Medicine, Wake Forest University School of Medicine, Winston-Salem, NC, USA; 4 Department of Exercise and Sport Science, East Carolina University, Greenville, NC, USA; 5 Center on Aging, Wake Forest University School of Medicine, Winston-Salem, NC, USA; 6 Institute of Anatomy and Musculoskeletal Research, Paracelsus Medical University, Salzburg, Austria, & Chondrometrics GmbH, Ainring, Germany; 7 Division of Research, New England Baptist Hospital, Boston, MA, USA

## Abstract

**Background:**

Obesity is the most modifiable risk factor, and dietary induced weight loss potentially the best nonpharmacologic intervention to prevent or to slow osteoarthritis (OA) disease progression. We are currently conducting a study to test the hypothesis that intensive weight loss will reduce inflammation and joint loads sufficiently to alter disease progression, either with or without exercise. This article describes the intervention, the empirical evidence to support it, and test-retest reliability data.

**Methods/Design:**

This is a prospective, single-blind, randomized controlled trial. The study population consists of 450 overweight and obese (BMI = 27–40.5 kg/m^2^) older (age ≥ 55 yrs) adults with tibiofemoral osteoarthritis. Participants are randomized to one of three 18-month interventions: intensive dietary restriction-plus-exercise; exercise-only; or intensive dietary restriction-only. The primary aims are to compare the effects of these interventions on inflammatory biomarkers and knee joint loads. Secondary aims will examine the effects of these interventions on function, pain, and mobility; the dose response to weight loss on disease progression; if inflammatory biomarkers and knee joint loads are mediators of the interventions; and the association between quadriceps strength and disease progression.

**Results:**

Test-retest reliability results indicated that the ICCs for knee joint load variables were excellent, ranging from 0.86 – 0.98. Knee flexion/extension moments were most affected by BMI, with lower reliability with the highest tertile of BMI. The reliability of the semi-quantitative scoring of the knee joint using MRI exceeded previously reported results, ranging from a low of 0.66 for synovitis to a high of 0.99 for bone marrow lesion size.

**Discussion:**

The IDEA trial has the potential to enhance our understanding of the OA disease process, refine weight loss and exercise recommendations in this prevalent disease, and reduce the burden of disability.

**Trial Registration:**

NCT00381290

## Background

Current therapy for knee osteoarthritis (OA) most often focuses on pain relief, often with nonsteroidal anti-inflammatory medications that have only modest functional benefit, do not slow disease progression, and have potentially serious cardiovascular and gastrointestinal side effects [1,2]. Recent evidence also casts doubt on the effectiveness of arthroscopic surgery for adults with mild-to-moderate knee OA [3]. Modern approaches to OA management should recommend targeting modifiable risk factors, including obesity, malalignment, and muscle weakness.

Obesity (BMI ≥ 30.0 kg/m^2^) is associated with increased risk of functional impairment and is considered the most modifiable risk factor for knee OA [4,5]. Although caloric restriction has produced dramatic changes in many biological systems and proven beneficial to obese adults suffering from knee OA, no study has directly addressed whether intensive dietary induced weight loss also slows the progression of structural damage in people with OA or determined mechanistically how it happens.

**Figure 1:**
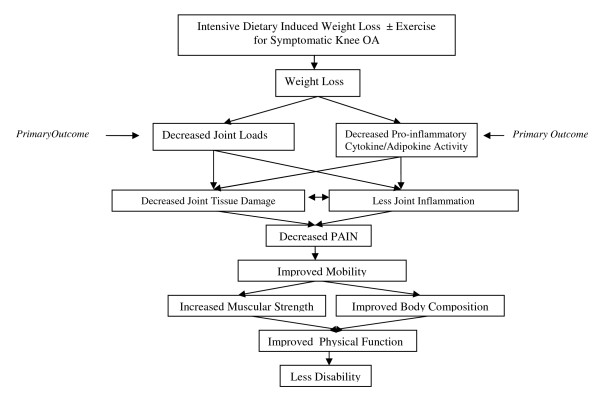
**Theoretical model by which intensive dietary restriction plus exercise decreases knee joint loads, improves strength and power, and decreases inflammation leading to a slowing of disease progression, reduced pain, improved function, and less disability**.

Dietary weight loss is considered the best potential non-pharmacologic intervention to prevent or to slow disease progression [6]. Christensen et al. [7] recently found that an 11% weight loss in older adults with knee OA over an 8-week period resulted in a 3-fold improvement in self-reported function relative to a control group. Radiographic evidence of progression was not studied in this trial and would not be expected to change in an 8-week period. The mechanisms responsible for improvements in function and pain in patients with knee OA consequent to long-term intensive dietary weight loss and exercise interventions in obese adults remain unknown. Reductions in joint loads and inflammatory cytokines, each thought to exacerbate joint destruction, are potential pathways. Our current Intensive Diet and Exercise for Arthritis (IDEA) randomized clinical trial will examine the effects intensive weight loss has on the biomechanical and inflammatory osteoarthritis disease pathways. We suggests that a long-term weight loss of at least 10% of baseline body weight, with or without exercise, will reduce knee joint loads and decrease inflammation resulting in a slowing of disease progression and improved clinical outcomes (Figure 1).

Recent studies demonstrate that low-grade inflammation plays a pathophysiological role in OA. The inflammatory cytokine interleukin-1 beta (IL-1β) is present in the joint fluids of OA patients [8]. An inflammatory component associated with OA can be detected in the circulation since serum concentrations of inflammatory markers, such as cytokines (interleukin-6, IL-6; tumor necrosis factor alpha, TNFα) and the acute-phase reactant C-reactive protein (CRP), are higher in persons with knee or hip OA compared to those without [9,10]. In addition, longitudinal studies demonstrate that high systemic CRP and TNFα concentrations predict increased radiographic progression of knee OA as much as 5 years later [10,11]. Moreover, OA severity and impaired physical function are associated with higher inflammatory markers in the blood [12]. Thus, severity, mobility, pain, stiffness, and radiographic progression may be partly mediated by the level of chronic inflammation in OA patients.

Obese individuals have higher concentrations of inflammatory markers and may be at greater risk of functional limitation and OA disease progression. Besides direct effects on the joint, inflammatory mediators can affect muscle function and sensitize nerves leading to increased pain [13]. Studies are beginning to show that both exercise training and weight loss decrease overall inflammation [14,15]. Our Arthritis, Diet, and Activity Promotion Trial (ADAPT) showed that a dietary intervention producing a 5% weight loss significantly reduced CRP, IL-6, and TNFα soluble receptor 1 concentrations compared to a control group [16]. We still do not know whether there is a "threshold" – a relative or absolute weight loss that maximally reduces inflammatory markers – or if improvements in physical function, pain, and OA progression are related to declines in chronic inflammation with weight loss and exercise.

Previous studies indicate that knee OA has an adverse effect on gait mechanics, but little is known about the influence of obesity. Epidemiological and biomechanical data suggest that the additional mechanical stress obesity places on weight-bearing joints increases the propensity for knee OA. We found that as BMI increased, older adults with knee OA changed their gait, walking slower and exerting greater impact forces [17]. There are no definitive data on whether weight loss is beneficial for reducing OA progression; however, these data support our proposed model in which intensive dietary induced weight loss and exercise interventions slow disease progression. This study could make intensive dietary weight loss combined with exercise the standard-of-care for overweight and obese adults with knee OA and enhance our understanding of the OA disease process and weight-loss and exercise recommendations for older people generally.

This paper describes the design of IDEA, a large scale long-term intensive weight loss and exercise randomized clinical trial, and provides new reliability data on knee joint loads calculated using musculoskeletal modeling, and measures of osteoarthritis disease progression using magnetic resonance imaging (MRI).

## Methods/Design

### Study Design

IDEA is a single-blinded, one center, 18-month, randomized controlled trial. Participants are randomized into one of 3 groups: exercise-only (E), intensive dietary induced weight loss (D), or intensive dietary induced weight loss-plus-exercise (D+E) (Table 1). Personnel responsible for data collection are blinded to group assignment. One-half of the participants are randomized to supplemental testing, including MRI, strength tests, and full-length x-rays of the lower extremities.

**Table 1:** Study Design

Intensive Dietary Restriction	Exercise	Intensive Dietary Restriction + Exercise
N = 150	N = 150	N = 150

### Study Sample

The study sample consists of 450 ambulatory, community-dwelling persons age ≥ 55 years with: (1) grade II-III (mild to moderate) radiographic tibiofemoral OA or tibiofemoral plus patellofemoral OA of one or both knees [18]; (2) 27.0 ≤ BMI ≤ 40.5 kg/m^2^; and (3) a sedentary lifestyle, defined as not participating in a program that incorporates more than 30 minutes per week of formal exercise within the past 6 months (Table 2). The study protocol was reviewed and approved by the Human Subjects Committee of Wake Forest University Health Sciences. Informed consent will be obtained from all study participants.

**Table 2:** Exclusion criteria

**Criteria**	**Exclusion**	**Method**
Significant co-morbid disease that would pose a safety threat or impair ability to participate, previous acute knee injury, patellofemoral OA in the absence of tibiofemoral OA	Symptomatic or severe coronary artery disease; severe HTN; active cancer, other than skin cancer; anemia; dementia; liver disease; COPD; peripheral vascular disease; inability to walk without an assistive device; blindness; osteoporosis, ligament or cartilage damage from acute event; type 1 diabetes; type 2 diabetes on thiazolidinediones agents; patellofemoral OA without tibiofemoral OA.	Medical history; physical exam; GXT; knee x-ray sunrise view
Ability and willingness to modify dietary or exercise behaviors	Unwillingness or inability to change eating and physical activity habits due to environment; cannot speak and read English	Questionnaire, assessment by interventionists
Excess alcohol use	≥ 21 drinks per week	Questionnaire
Inability to finish 18-month study or unlikely to be compliant	Lives > 50 miles from site or planning to leave area ≥ 3 months during the next 18 months	Questionnaire
Conditions that prohibit knee MRI	Pacemaker, severe claustrophobia, defibrillator, implanted metal objects in leg, neurostimulator, magnetic aneurysm clip, any kind of metal implant or foreign metal objects in the body, such as bullets, shrapnel, metal slivers	Medical history
Significant cognitive impairment or depression	diagnosis of dementia or a Modified Mini-Mental State exam (3MSE) score < 70, CES-D score > 17	Medical history, 3MSE, CES-D

These criteria aim to assure that we include people who will benefit from intensive dietary restriction [19]. By enrolling overweight and obese people with a BMI no greater than 40.5 kg/m^2^, we enhance the likelihood that we will achieve our goal of moving each participant down one BMI class (from Class II to Class I obesity [BMI < 35 kg/m^2^], Class I obesity to overweight [BMI between 27–30 kg/m^2^], or overweight to normal [BMI < 25 kg/m^2^]) [20]. Since BMI categories carry different risks for diseases and mortality, by reducing them one full category, more profound effects on disease markers may reasonably be expected. During the study, all participants are allowed to maintain their usual medications, including NSAIDs and other analgesics. With their physician's consent, they can also reduce medication if pain decreases. A detailed record of medication use is collected at baseline and 6- and 18-month follow-up testing.

### Interventions

#### Intensive dietary induced weight loss intervention

Both the D and D+E groups receive the same dietary intervention. Our weight loss goal is a group mean of at least 10% of baseline weight, with a desired 15% weight loss. The dietary plan is based on partial meal replacements, including up to 2 meal-replacement shakes per day (provided by General Nutrition Centers, Inc., Pittsburgh, PA.). All participants are closely monitored, and their daily caloric intake adjusted according to their rate of weight change.

Initial diet plans provide an energy-intake deficit of 800–1000 kcals/day as predicted by energy expenditure (estimated resting metabolism × 1.2 activity factor). The lowest calorie level permitted is 1100 kcals for women and 1200 kcals for men. The calorie distribution goal is 15–20% from protein, < 30% from fat, and 45–60% from carbohydrates, consistent with the Dietary Reference Intakes for Energy and Macronutrients [21] and successful weight-loss programs [22]. For the third meal, participants follow a weekly menu plan and recipes provided by the study composed of traditional foods. It contains 500–750 kcals and is low in fat and high in vegetables. Snacks include a snack bar, fruit, or vegetable providing approximately 100–120 kcals each. The food plan is tailored to individual preferences and energy needs. Meal plans are developed by the intervention staff to provide the prescribed macronutrient-balanced energy intake as fewer meal replacements are consumed during the trial.

Body weight is monitored in weekly nutrition education and behavioral sessions. Nutrition interventionists, trained in behavioral therapy and experienced in working with older adults, run all group and individual sessions. There is one individual session and 3 group sessions per month for the first 6 months. The behavioral sessions focus on awareness of changing eating habits to lower caloric intake. Information on what food changes to make, how to make them, and why they are important is clearly explained and discussed with participants and significant others. Group sessions include problem solving, review of a specific food topic, and tasting of several well-balanced, low-fat, nutritious foods prepared with easily available ingredients. During the individual sessions, the nutrition interventionist reviews individual progress, solves problems, answers questions, and sets goals. From months 7 to 18, participants attend biweekly group sessions, with an individual appointment every 2 months.

Once they reach the study's desired weight-loss goal of 15% of baseline body weight, participants either begin weight maintenance or continue to lose weight using safe, healthy nutritional practices. Weight loss can continue throughout the intervention, provided the individual wants to and has not reached a level that may be associated with health hazards in this population; i.e., a >20% body weight loss at 6 months or > 30% at 12 months. Weight maintenance uses relapse-prevention techniques that include managing the environment, adjusting appropriate goal-based daily energy needs, and continuing self-regulatory skills.

For participants who have difficulty achieving the weight-loss goal, we use a behavioral "toolbox" that includes frequent individual behavioral counseling sessions, incentives, home-assessments, or other items deemed appropriate. Diet plans can be altered to restrict one of the snacks and/or the size of the evening meal. Participants record their food and beverage intake in daily logs that are collected monthly and used as a self-monitoring tool for dietary intake. Nutrition interventionists use them to design rescue procedures, using behavioral and educational strategies.

#### Alert Values

Weight is monitored weekly. A loss of >20% body weight after 6 months or >30% after 12 months triggers a safety alert.

#### Exercise intervention

The exercise intervention is identical for both exercise groups. Sixty-minute sessions are conducted 3 d·wk^-1 ^for 18 months, and each group (E and D+E) exercises at a different time to prevent crossover effects from their interactions. The first 6 months are facility-based. Anytime after 6-month follow-up testing is completed, participants that would like to add home based exercise to their training routine will alternate between the facility (2 d·wk^-1^) and home (1 d·wk^-1^) during a 2-week transition phase. Subsequently, participants can decide to remain in the facility program; opt for a home-based program; or combine the facility and home-based programs.

The 3 d·wk^-1 ^exercise program consists of an aerobic phase (15 min), a strength training phase (20 min), a second aerobic phase (15 min), and a cool-down phase (10 min). The strength-training phase is particularly relevant to help offset any possible loss of muscle and bone mass resulting from dietary weight loss.

Walking is the primary mode of aerobic training. Participants who meet the inclusion criteria but cannot walk for extended periods use upright and recumbent stationary bicycles to supplement walking. Exercise intensity ranges between 50–75% of the heart-rate reserve, using the symptom-limited maximum heart rate obtained from a graded exercise test (GXT). The strength training session is approximately 20 minutes and targets lower extremity muscles and, to a lesser extent, upper body muscles. This phase includes 1–2 sets of 10–12 repetitions on leg extension, leg press, seated leg curl, seated calf raise, compound row, and vertical chest or incline press.

During months 7–18, home-based participants are telephoned every other week in the first 2 months, every third week during the next 2 months, and every month thereafter. Monthly exercise and attendance logs are gathered to monitor progress. The home-based participant exercises at home 3 d·wk^-1 ^and comes to the facility once a month for an individual meeting with the exercise interventionist. Home-based strength training utilizes a Thera-Band exercise program.

#### Intensive dietary induced weight loss-plus-exercise intervention

Participants randomized to the D+E intervention receive both interventions described above. Dietary counseling is scheduled before or after the exercise sessions to minimize the number of visits.

#### Techniques to Improve Adherence

We conservatively estimate 80% retention and 65% adherence rates over this 18-month intervention. Adherence to the nutrition intervention is defined as attending the nutrition classes; to the exercise sessions, the number of sessions completed divided by the number scheduled (72 weeks × 3 d·wk^-1 ^= 216 sessions).

The health psychologist trains the exercise interventionists in behavioral techniques that include: (1) tailoring training to the participant's ability; (2) frequent contact during all intervention phases; (3) clear behavioral goals and feedback on achievements; (4) training participants to self-monitor their heart rate and to complete activity logs; (5) establishing personal commitment to the project through the exercise leader; and (6) targeting educational materials and field trips to prevent relapse and to develop prompts to exercise. Primary interventionists regularly review adherence data and identify any participants who need additional reminders and/or counseling.

### Trial Conduct

#### Recruitment

The 30-month recruitment period is divided into 10 waves of 45 participants each, entering the study at 3-month intervals. Recruitment strategies include mass mailings, newspaper advertisements, and presentations at local aging service networks, senior centers, and churches. Our Claude D. Pepper Older Americans Independence Center recruitment core also has access to a large database of older adults who have consented to be contacted about participation in clinical trials. Selected physicians in orthopedics, geriatrics, and rheumatology receive information on the study and encourage qualified patients to contact IDEA recruitment personnel for further information. Specific strategies aim to maximize the number of African Americans who qualify for, and are enrolled in, the study. At biweekly meetings, all recruitment activities and the number of participants randomized are reviewed.

#### Screening (SV) and follow-up (FU) visits

**Figure 2:**
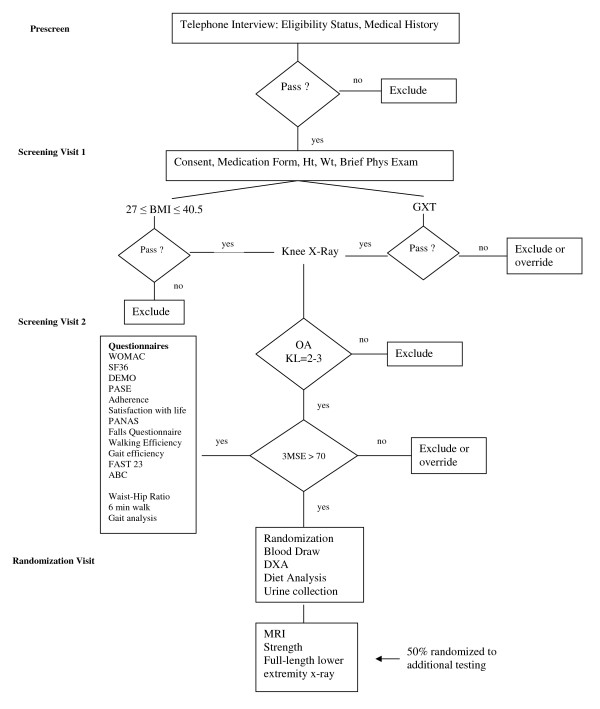
**Participant eligibility and screening**.

Figure 2 shows eligibility and screening measurements for the IDEA trial. All participants are tested at baseline and 6 and 18 months post randomization. A subset of 225 participants receive supplemental performance tests, x-ray, and MRI (Figure 2).

### Measurements

#### Screening measurements

The Eligibility Questionnaire addresses joint pain, physical function, activity level, co-morbid diseases, willingness to participate in an 18-month study, height and weight (to determine BMI), caregiver status, status of significant others, and distance of home from the center (within a 50-mile radius). Demographic information includes data on occupation, income, and education. The medical form is used to determine co-morbidities and to analyze medication data gathered by self-report. A computerized system places individual drugs in major therapeutic categories to assess changes in nonsteroidal anti-inflammatory drug use. Participants undergo an initial symptom-limited, maximum exercise stress test to exclude anyone who has severe manifestations of coronary heart disease using a modified Naughton protocol that has 10 stages. Volunteers are screened for cognitive deficiencies using the Modified Mini-Mental State Exam (3MSE) [23]. A score of < 70 justifies exclusion because we feel that cognitively impaired persons could not adhere to the protocol, especially the dietary requirements for the weight-loss groups.

#### Inflammatory Markers

We plan to assess biological markers of inflammation before and after weight-loss and exercise treatments to determine whether they significantly lower inflammation levels. Our primary measure is IL-6. This cytokine is consistently implicated in OA pathogenesis and showed significant improvement with weight loss in ADAPT. We will also measure CRP as an overall marker of chronic inflammation [10] and TNFα and TNFα soluble receptor 1 (sTNFR1) because these cytokines are also implicated in OA pathogenesis [24]. Leptin, an adipokine that increases synthesis of TGFβ, a known stimulator of osteophyte formation, is also measured.

#### Blood and Urine Sample Collection

Blood samples (approximately 40 ml per visit) for assessing biomarkers are collected via venipuncture in the early morning, after a 12-hour fast and at baseline, 6-month, and 18-month assessment visits. Urine samples (20 ml per visit) are collected in 250 ml specimen cups by each participant for analysis of new and emerging OA biomarkers. In the event of acute respiratory, urinary tract, or other infection, we postpone blood and urine sampling for 1–2 weeks after recovery from symptoms.

#### Gait Analysis

Our primary measures of knee joint loads are tibiofemoral compressive forces and the internal abduction moment. We obtain 3-D kinematic gait data using a 25-reflective marker set, arranged in the Cleveland Clinic configuration, a 6-camera Motion Analysis System sampling at 60 Hz and EVaRT 4.4 software. Ground reaction forces and moments are obtained with a 6-channel force platform (Advanced Mechanical Technologies, Inc., Newton, MA) operating at 480 Hz. Six successful trials are collected on each participant, and 3 chosen for subsequent analysis. A successful trial is defined as one in which the participant's entire foot is placed on the surface area of the force platform while walking within ± 3.5% of freely chosen speed.

A Butterworth low-pass filter with a 6 Hz cut-off frequency is used to remove high frequency noise from the linear kinematic data. OrthoTrak 6.0 β4 clinical gait analysis software is used to generate lower extremity angular kinematic and kinetic data using inverse dynamics. Knee-joint forces are calculated using a model developed by DeVita et al[25,26]. We have successfully applied it to healthy and OA subjects[25,26], finding ranges (2.8 – 6.0 BW) similar to those in previous studies [27,28].

#### Test-retest reliability and the effect of BMI

**Figure 3:**
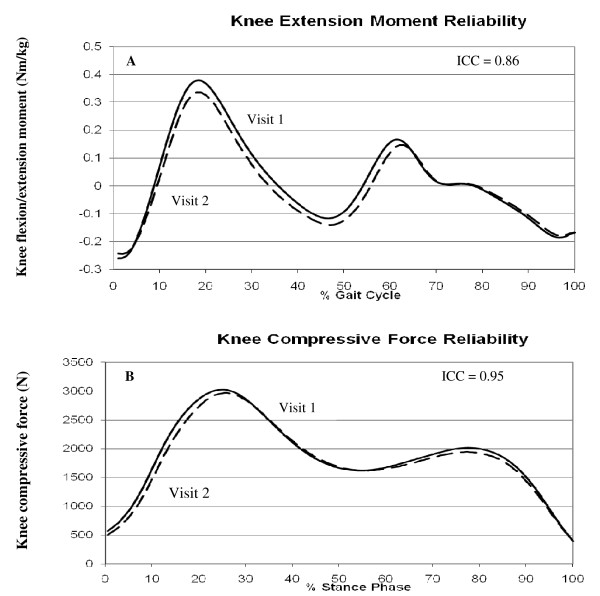
**Test-retest reliability for knee joint loads calculated during gait**. (A) Knee flexion/extension moment with an ICC = 0.86, and (B) Knee compressive forces with an ICC = 0.95.

The reliability of the gait data was determined using 21 IDEA participants (4 males, 17 females) with a mean age of 65.7 ± 5.8 yrs, a mean mass 92.4 ± 13.0 kg, and a mean height of 165.6 ± 10.0 cm. Participants were tested twice, 1 week apart. Intra-class correlation coefficients (ICC) were used to compare the between day reliability of peak internal knee flexion/extension and internal knee abduction/adduction moments, peak knee compressive force, peak vertical ground reaction force, and stride length. Overall day-to-day reliability was excellent with ICC values ranging from 0.86–0.98 for the selected variables (Table 3). Knee flexion/extension moment and stride length were most affected by BMI, with lower reliability associated with the highest tertile of BMI (Table 3 and Figure 3).

**Table 3:** Test-retest reliability (ICC) of selected gait variables for all subjects (n = 21) and in tertiles of BMI (kg/m^2^)

Gait variable	All Subjects	34 < BMI ≤ 41	32 ≤ BMI ≤ 34	27 ≤ BMI < 32
Knee Extensor Moment	0.86	0.80	0.72	0.94
Knee Abductor Moment	0.94	0.95	0.97	0.86
Knee Compressive Force	0.95	0.90	0.95	0.98
Vertical GRF	0.98	0.92	0.95	0.99
Stride length	0.95	0.69	0.97	0.99

#### Western Ontario McMasters Universities Osteoarthritis Index (WOMAC)

The WOMAC is used to measure self-reported physical function and pain [29]. The LK version asks participants to indicate on a scale from 0 (none) to 4 (extreme) the degree of difficulty experienced performing activities of daily living in the last 48 hours due to knee OA. Individual scores for the 17 items are totaled to generate a summary score that can range from 0–68, with higher scores indicating poorer function.

The pain index assesses participants' pain on the same scale, ranging from 0 (none) to 4 (extreme). The pain subscale consists of 5 items and total scores can range from 0–20, with higher scores indicating greater pain. This instrument is recommended by the Osteoarthritis Research Society as the health status measure of choice for older adults with knee OA. It has been validated for use in orthopaedic and pharmacologic interventions [29,30].

#### Mobility

Our mobility measure is 6-minute walk distance. Participants are told to walk as far as possible in 6 minutes on an established flat, indoor course. Six-minute walk distance is related to symptom-limited maximal oxygen consumption (r = 0.53) and the test has a 3-month test-retest reliability of 0.86 [31].

#### Body Composition

Body mass index is calculated as mass (measured in kilograms on a standard calibrated scale) divided by height squared (measured in meters). Whole body lean mass (LM), fat mass (FM), and bone mass are measured by DXA on all 450 participants. BMD is also measured at the posteroanterior (PA) lumbar spine and hip. DXA scans are obtained with a fan-beam scanner, Delphi A™, Hologic (Waltham, MA), using manufacturer's recommendations for patient positioning, scan protocols, and scan analysis. Coefficients of variation (%CV) are 1.2% for whole body FM; 0.5% for whole body LM; 0.9% for whole body BMD; 1.2% for PA spine BMD; and 0.9% for total hip BMD.

#### Strength

We measure bilateral knee concentric strength at baseline, FU6, and FU18 on a random sample of participants (n = 225) with equal numbers in each group using a Kin-Com 125E isokinetic dynamometer set at an angular velocity of 30 deg/sec. Grip strength, an excellent measure of overall body strength, is also measured using a standard grip strength dynamometer.

#### Medications

During SV1 and FU visits, participants are administered a medication questionnaire adapted from the Atherosclerosis Risk In Communities (ARIC) study [32] and widely used in field research and our prior studies. We obtain information about all prescription and over-the-counter medicines and supplements used during the 2 weeks prior to the interview.

#### MRI

MRI of the most symptomatic knee will be performed at baseline and FU18. MRI scans are performed with 1.5 Tesla General Electric scanners using an extremity coil. The following MR sequences were obtained: (1) Double oblique coronal three-dimensional spoiled gradient echo (SPGR) with fat suppression; (2) Axial T1-weighted spin-echo (SE); (3) Double oblique coronal T1-weighted SE; (4) Sagittal T1-weighted SE; (5) Sagittal T2-weighted fast spin echo (FSE) with fat suppression; (6) Double oblique coronal T2-weighted fast spin echo (FSE) with fat suppression.

#### Semi-quantitative scoring of whole joint

MRI has the capability of visualizing all potentially relevant OA joint structures; therefore, it is not surprising that it is an important tool in improving our understanding of knee OA by providing a non-invasive tool for the study of healthy and diseased states, as well as in providing a means of assessing risk factors for and the effectiveness of therapeutic interventions for prevention of pain, dysfunction, and disability in OA. MRI scoring will be read paired and blinded to timepoint by a musculoskeletal radiologist using the BLOKS method [33]. The following MRI features are scored: (1) Bone marrow lesion (BML) size; (2) BML % area; (3) Percent BML rather than cyst; (4) Cartilage loss; (5) Extent of any cartilage loss at specified points;(6) Osteophytes; (7) Synovitis; (8) Effusion; (9) Meniscal extrusion; (10) Meniscal signal; (11) Meniscus tear; (12) Ligaments: presence or absence of tear in patella tendon, ACL or PCL; and (13) Periarticular features: presence or absence of abnormal features such as pes anserine bursa and iliotibial band.

Test-retest reliability was performed on 10% of scans (N = 22) 1 week apart. Prior data demonstrates the inter-reader reliability for the BLOKS items range from 0.51 for meniscal extrusion up to 0.79 for meniscal tear. The reliability for other key features is 0.72 for BML grade, 0.72 for cartilage morphology, and 0.62 for synovitis [33]. Our reliability exceeded previously reported results (Table 4). Reliability for meniscal extrusion was 0.95; meniscal tear, 0.99; BML size, 0.72, cartilage morphology, 0.93, and synovitis 0.66.

**Table 4:** Intra-observer reliability for reading of MRI BLOKS features (weighted kappa)

**BLOKS feature**	**Reliability** **(weighted kappa)**
BML size	0.72
BML % area	0.67
% of lesion BML	0.79
Cartilage 1 morphology	0.95
Cartilage 2	0.93
Osteophyte	0.74
Synovitis	0.66
Effusion	0.77
Meniscal extrusion	0.95
Meniscus tear/cysts	0.99

BLOKS = Boston Leeds Osteoarthritis Knee Score

BML = Bone Marrow Lesion

#### Quantitative Cartilage Morphometry

**Figure 4:**
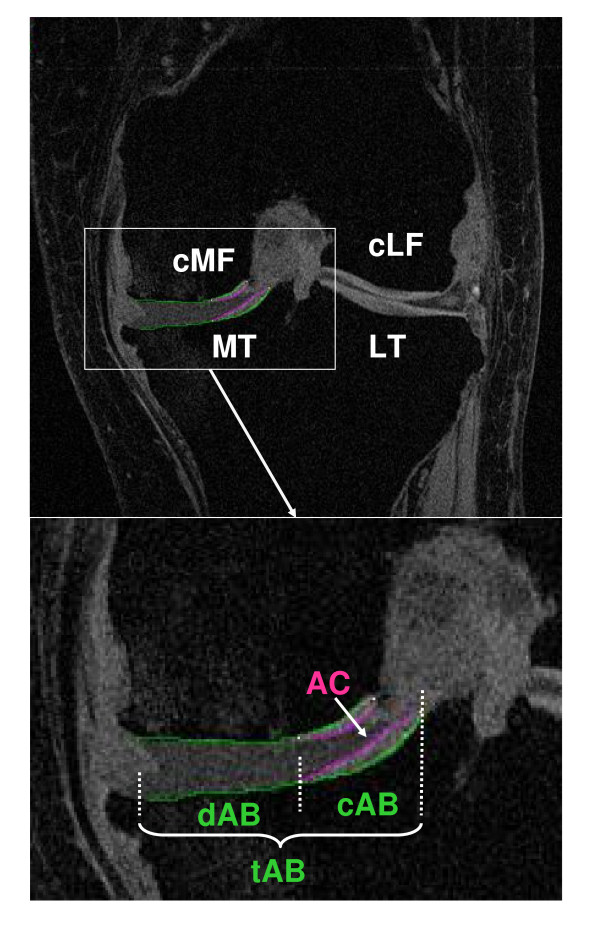
**Baseline double oblique coronal MR image acquired using a fat suppressed SPGR sequence with a 1.5 mm slice thickness and 0.31 mm × 0.31 mm in plane resolution**. The area of the cartilage surface (AC) and the total subchondral bone area (tAB) are manually segmented in the medial tibia (MT) and weight-bearing medial femur (cMF). The part of the tAB covered by AC is defined as the cartilaginous area of bone (cAB), that not covered by the AC as the denuded area of bone (dAB).

**Figure 5:**
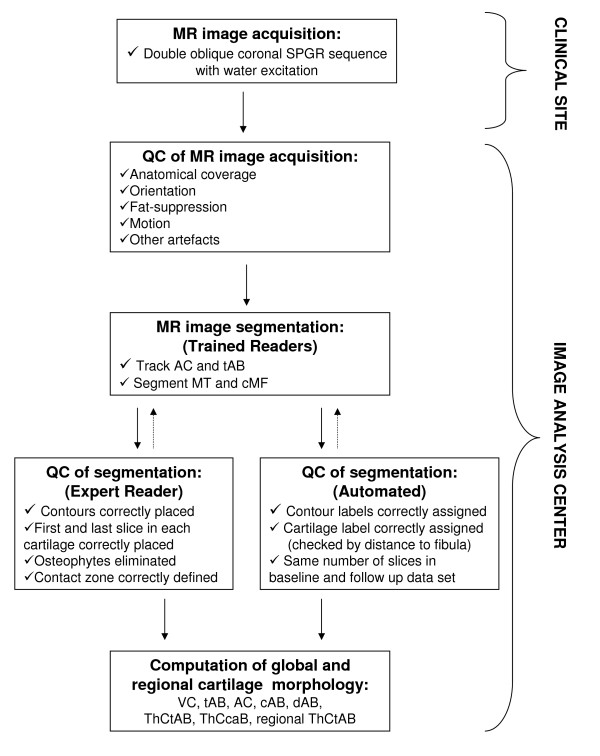
**Flow chart visualizing the image analysis and quality control (QC process)**.

Measurements are confined to the medial femorotibial compartment, because (1) it is the most common site of knee OA [34]; (2) OA there is strongly related to bone marrow edema lesions and varus knee alignment [34,35]; and (3) we have additional, well-established measures of joint integrity using BLOKS [33]. The tibia (MT) and central weight-bearing medial femur (cMF) are segmented by manually tracing of the total area of the subchondral bone (tAB) and the area of the cartilaginous joint surface (AC) on a slice-by-slice basis [36,37]. Segmentation of tAB included all areas of cartilage-covered and denuded subchondral bone, but not osteophytes [36,37]. Likewise, AC segmentation excluded osteophyte cartilage. MT was segmented throughout all slices that displayed cartilage; analysis of the femoral condyle was confined to the portion of the condyles that is displayed without relevant partial volume effect in coronal images (cMF). All segmented data are quality controlled (QC'd) by one expert by checking all segmented slices of each data set, and corrections of the segmentation were performed if necessary (Figures 4 &5).

**Figure 6:**
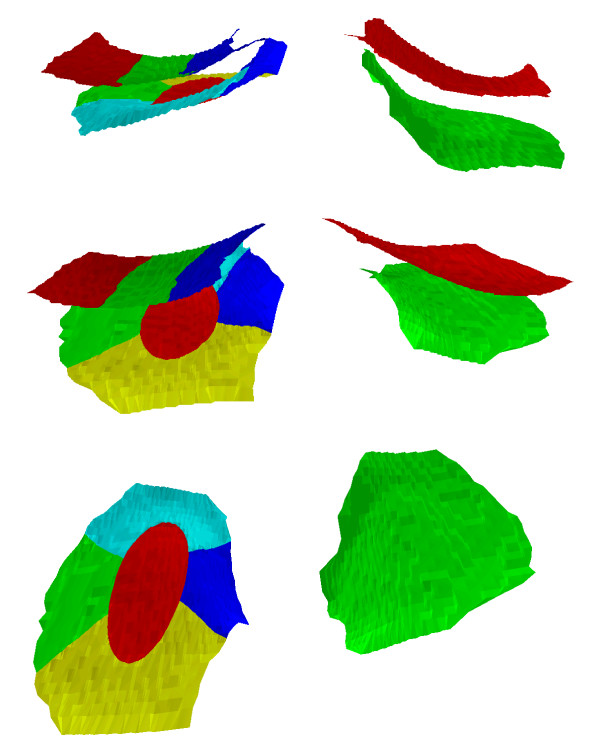
**3D visualization of the femorotibial subregions: Top: anterior view with the medial tibia being divided into 5 subregions (red = central, green = external, dark blue = internal, yellow = posterior, and turquoise = anterior subregion**. The central subregion is defined by a cylinder and occupies 20% of the total subchondral bone area of medial tibia (MT). The weight bearing femur is divided into three strip-like subregions, each occupying 33.3% of the subchondral bone area (green = central, dark blue = internal, red = external subregion). The total subchondral bone area of the lateral tibia is shown in green and that of the lateral femur in red. Middle: oblique anterior-superior view of the subregions. Bottom: superior view onto the tibial subregions (femur not shown).

The same software used for segmentation and QC readings is used to calculate the size of the total area of the subchondral bone (tAB), the tAB covered with cartilage (cAB), the denuded bone area (dAB), the area of the cartilage surface (AC), the mean cartilage thickness across the entire tAB (ThCtAB), counting all denuded areas as 0 mm cartilage thickness the mean cartilage thickness across cAB (ThCcAB), and maximal cartilage thickness. In addition, subregional measurements are performed by analyzing ThCtAB in central, internal, external, anterior and posterior subregions of MT, and central internal and external subregions of cMF, using a recently developed approach by Wirth et al. [38]. Figure 6 represents a 3-D visualization of the segmented data.

### Health-Related Quality of Life

The SF-36 [39] is the most widely used and carefully validated measure of HRQL. It yields two broad summary scores: physical health and mental health.

### X-ray

To assess lower limb alignment, a full-length anteroposterior radiograph (AP) of each lower extremity is obtained at baseline on a random sample (n = 225) with equal numbers in each group. Subjects are positioned following the methods of Sharma et al. [40]. Alignment is defined as the measure of the angle formed by the intersection of the line connecting the centers of the femoral head and intercondylar notch and connecting the centers of the ankle talus and tibial spines. A varus knee is an angle > 0° in the inward direction, and valgus is an angle > 0° in the outward direction.

Bilateral, anterior-posterior, weight-bearing knee x-rays are used to identify tibiofemoral arthritis, and sunrise views to identify patellofemoral OA. Anterior-posterior x-rays are repeated at FU18 to assess changes in joint-space width and Kellgren and Lawrence (K-L) score. MRI is sensitive in detecting changes in joint structure and is the primary measure for progression in this trial. X-rays are evaluated because (1) current regulatory guidance recommend them as the gold standard for measuring disease progression ; and (2) obtaining plain films of both knees to monitor disease progression bilaterally is straightforward.

Participants' knees are flexed at 15 degrees using a positioning device, and the beam centered on the joint space. The x-ray beam is directed perpendicular to the cassette to pass between the femoral condyles and the patella surfaces. The exact angle of the beam depends upon the degree of flexion of the knee and the participant's individual body habitus. The focus-to-film distance is held constant throughout the study. This method of acquisition standardizes positioning to optimize reproducibility [41] but may not afford the same responsiveness of fluoroscopic methods of acquisition [42]. A single physician, masked to the treatment group, will read the radiographs. Severity of tibiofemoral OA is measured using the K-L grading scale and defined as present if K&L grade is ≥ 2 [18]. The minimum joint-space width of both compartments is measured using a 0.1 mm graduated magnifying lens to assess disease progression

### Modified Mini-Mental State Exam (3MSE)

The 3MSE is an objective, quantitative assessment of cognitive functioning that examines orientation to time and place, recall, short-term memory, and arithmetic ability. Participants scoring below 70 are not eligible for the study.

### Physical Activity Scale for the Elderly (PASE)

The PASE scale, used to estimate activity levels, has proven reliable in many of our clinical trials [43].

### Accelerometry

Physical activity monitoring is also performed using accelerometry on the same random sample of 225 participants. Participants are provided a Lifecorder EX uniaxial accelerometer at baseline, 6, and 18-month follow-up and asked to wear it for 7 consecutive days. It measures step counts, daily energy expenditure, physical activity patterns, and daily activity energy expenditure.

### Dietary Assessment

Dietary intake will be assessed at our General Clinical Research Center (GCRC) by registered dietitians who are not involved in the intervention. Participants complete a 3-day food record at baseline, 6, and 18 months, which is analyzed for nutrients using the accurate, standardized, and comprehensive Minnesota Nutrition Data System (NDS). Portion sizes are estimated using 2D food-portion visuals available from NDS and discussed with the participant at baseline.

### 2.4.19. Body weight, height, BMI, waist circumference

Body weight, height, and waist circumference are obtained at baseline, 6, and 18 months using standard techniques. The range of BMIs for inclusion is 27.0 to 40.5 kg/m^2^.

### Statistical Considerations

#### Statistical Analyses

All primary analyses will be based on the intention-to-treat method in which each participant is included in the initial randomization group regardless of adherence. IDEA stratification factors, BMI, and gender will be included in all statistical models, so the analysis will match the design, and the estimated variance will not be biased. Assumptions will be verified for all models, and appropriate transformations used when necessary.

#### Primary Aim

The primary aim of this study is to test whether the intensive dietary intervention, the exercise-only intervention, and a combination affect inflammation (IL-6) and knee joint loads (knee maximum compressive force) differently. Analyses for the primary aim will test the effect of the 3 interventions on the primary outcome measures using repeated measures analysis of covariance (ANCOVA). These methods are robust-to-moderate departures from linearity when the covariates are not extremely different. Eighteen months after randomization, intervention effects will be estimated and tested, adjusting for prerandomization levels, BMI, and gender. Subsequent models will be adjusted for other variables, including age, education, long-term individual preferences for exercise, and specific factors showing group differences at baseline.

#### Secondary Aims

The effect of the interventions on WOMAC function and pain; inflammation markers, including CRP, TNF-α, TNF-sR1, and leptin; and maximal internal abduction moment will be determined with repeated measures ANCOVA and statistical tests and estimates as described above, verifying assumptions and using appropriate transformations. We will examine whether increased weight loss slows disease progression and how changes in the biomechanical and inflammatory pathways result in improved function, diminished pain, and slower disease progression. Multivariate and multiple regression methods will be used to study the associations between factors in the hypothesized pathway.

#### Sample size calculations

A total sample size of 450 (150/group) provides 80% statistical power to detect 20% differences in IL-6 and 15% differences in knee joint loads at the two-sided 0.008 significance level (adjusted for 3 pairwise intervention comparisons and 2 outcomes) with 80% retention. Our preliminary data suggest that a 20% difference in IL-6 is clinically important, and an additional 5% weight loss for a 200 lb patient would result in a 120 lb difference in knee joint loads. Estimates of mean squared error were obtained from the ADAPT study [44], which measured the same outcomes and used a similar patient population. Based on the assumptions above and the evaluation of secondary outcomes, the trial is designed to ensure that 120 subjects per group are evaluated at the end of 18 months.

## Discussion

Finding interventions that will slow or stop disease progression is a critical area of OA research. Pharmacologic and surgical interventions have had limited success [3,45], and nonpharmacologic interventions, such as exercise and dietary induced weight loss, improve self-reported physical function and pain but appear to result in similar disease progression rates as attention control groups [44,46]. Modest weight loss is a recommended treatment for knee OA [47]; however, a 5% weight loss, alone or in combination with exercise, does not attenuate disease progression [44]. IDEA is the first adequately powered, long-term trial to examine whether more intensive weight loss, with or without exercise, will reduce inflammation and knee joint loads sufficient to alter disease progression.

Weaknesses of the study include the challenges involved in losing and then maintaining significant weight loss, and the lack of a gold-standard biomechanical model to estimate knee joint loads. Our previous work suggests that long-term intensive weight loss is required to affect the OA disease process. However, achieving long-term weight loss in obese individuals is challenging. Interventions lasting longer than 1 year that attain at least a 10% weight loss are rare. Esposito et al. [22] produced a 14.7% loss in body weight over a 2-year period in women following a moderate energy restricted diet (1300 kcals year 1 and 1500 kcals year 2). We will incorporate partial meal replacements, frequent contacts, and an intensive behavioral intervention that emphasizes self-regulatory skills and relapse prevention.

Musculoskeletal models only provide estimates of actual knee joint biomechanics. The principal limitation of our model is the absence of most knee ligaments. This will increase knee-muscle force predictions, since these tissues must resist all external loads. The model does include the lateral collateral ligament, which produces the principal non-muscular restraint during the stance phase of walking. We have successfully applied it to healthy and OA subjects [25,26,48], finding ranges similar to those of previous studies [27,28].

In addition to the benefit and feasibility of intensive weight loss in this population, IDEA will elucidate the benefits of exercise, both with and without intensive weight loss, on knee joint loads, stability, and neuromuscular function. The Osteoarthritis Research Society International (OARSI) has strongly recommended exercise as part of the standard of care for patients with knee OA [49]. Our previous work indicated that long-term exercise improves walking mechanics and reduces knee pain [46,50]; however, to our knowledge, no studies have examined the effects of exercise on knee joint loads in this population. Data from animal models suggest that the magnitude and direction of the applied loads influence whether knee articular cartilage responds favorably to regular exercise [51]. Using qMRI, IDEA will determine *in vivo *the relationship between knee joint loads and cartilage thickness changes. MRI may also detect effects of weight loss and exercise on OA progression because it is more sensitive to change than traditional radiographs.

Knee alignment is a known risk factor for disease progression [40]. Several authors have suggested that obesity exacerbates disease progression only in the presence of mild varus knee alignment [34]. As a secondary outcome, we will examine baseline knee alignment to determine its influence on 18-month disease progression using both semi-quantitative (BLOKS) and quantitative MRI (qMRI).

The IDEA trial has substantial potential to enhance our understanding of the OA disease process, refine weight-loss and exercise recommendations in this prevalent disease, and to ultimately reduce the burden of disability in our aging population. This study could make intensive weight loss with exercise the standard-of-care for overweight and obese adults with knee OA, as it enhances our understanding of the OA disease process and weight-loss and exercise recommendations for older people generally. Moreover, the use of MR imaging may increase our capability to detect effects of weight-loss and exercise on OA progression because of its higher sensitivity to change compared with x-ray.

## Competing interests

The authors declare that they have no competing interests.

## Authors' contributions

SPM conceived the study, participated in its design and coordination, carries out the biomechanical gait and strength analysis, and drafted the manuscript. CL participated in its design, coordinates statistical analyses and data management. SM participated in its design, and coordinates patient compliance and adherence protocols. GDM participated in its design, and carries out the nutrition intervention. RFL participated in its design, and coordinates x-ray readings, and carries out osteoarthritis biomarker analysis. PD participated in its design, helps coordinate the biomechanical gait analysis, and musculoskeletal modeling. ML carries out the patient evaluation of GXT results, and makes decisions regarding inclusion/exclusion based on medical tests and medical history. FE carries out the qMRI readings. DJH participated in its design and coordination, and carries out the MRI BLOKS analysis. JDW participated in its design and coordination, and is the medical director of the trial. BJN participated in its design and coordination, and carries out the biomarker analyses. All authors read and made comments on previous drafts of the manuscript, and approved the final manuscript.

## Pre-publication history

The pre-publication history for this paper can be accessed here:


